# Consistent size-independent harvest selection on fish body shape in two recreationally exploited marine species

**DOI:** 10.1002/ece3.1075

**Published:** 2014-05-01

**Authors:** Josep Alós, Miquel Palmer, Marta Linde-Medina, Robert Arlinghaus

**Affiliations:** 1 Instituto Mediterráneo de Estudios Avanzados, IMEDEA (CSIC-UIB)C/Miquel Marqués 21, 07190, Esporles, Illes Balears, Spain; 2 Department of Biology and Ecology of Fishes, Leibniz-Institute of Freshwater Ecology and Inland FisheriesMüggelseedamm 310, 12587, Berlin, Germany; 3 Faculty of Life Sciences, University of ManchesterM13 9PT Manchester, U.K.; 4 Chair of Integrative Fisheries Management and Integrative Research Institute for the Transformation of Human-Environment Systems (IRI THESys), Faculty of Life Sciences, Humboldt-Universität zu BerlinInvalidenstrasse 42, 10115, Berlin, Germany

**Keywords:** Behavior, ecomorphology, fisheries-induced selection, geometric morphometrics, predator–prey interactions, recreational fishing

## Abstract

Harvesting wild animals may exert size-independent selection pressures on a range of morphological, life history, and behavioral traits. Most work so far has focused on selection pressures on life history traits and body size as morphological trait. We studied here how recreational fishing selects for morphological traits related to body shape, which may correlate with underlying swimming behavior. Using landmark-based geometric morphometrics, we found consistent recreational fishing-induced selection pressures on body shape in two recreationally exploited marine fish species. We show that individuals with larger-sized mouths and more streamlined and elongated bodies were more vulnerable to passively operated hook-and-line fishing independent of the individual's body size or condition. While the greater vulnerability of individuals with larger mouth gapes can be explained by the direct physical interaction with hooks, selection against streamlined and elongated individuals could either involve a specific foraging mode or relate to underlying elevated swimming behavior. Harvesting using passive gear is common around the globe, and thus, size-independent selection on body shape is expected to be widespread potentially leaving behind individuals with smaller oral gapes and more compact bodies. This might have repercussions for food webs by altering foraging and predation.

## Introduction

Hunters and fishers nonrandomly harvest animals based on the traits they carry, which results in phenotypic and potentially genetic changes (Allendorf and Hard 2009). In many cases, larger-bodied individuals are preferentially captured and removed from the population, which may evolutionarily alter life histories and have repercussions for recovery, catchability, and yield (Jørgensen et al. 2007; Laugen et al. 2014; Alós et al. in press). The consequences of fisheries-induced direct or indirect selection on behavioral traits are less understood, but it may be also relevant under certain situations (Heino and Godø 2002; Uusi-Heikkilä et al. 2008). For example, there is increasing evidence that the odd of catching fish with passively operated gears, like hook-and-line, increases with swimming activity, space use, and in some species with risk-taking behavior (boldness) (Alós et al. 2012, in press; Biro and Post 2008; Heino and Godø 2002; Klefoth et al. 2012, 2013; Olsen et al. 2012; Sutter et al. 2012; but see Wilson et al. 2011). In fact, changes in life history traits like boldness may emerge from direct selection on behavioral traits in some species (Biro and Post 2008; Uusi-Heikkilä et al. 2008; Alós et al. in press). Compared to selection studies in terms of life history and behavior, limited studies exist that have examined selection pressures acting on morphological traits other than body size. However due to the often higher heritability of morphological traits compared to life history or behavioral traits (Mousseau and Roff 1987), selection on body shape may lead to rapid evolution of low-vulnerability morphotypes in response to fishing-induced selection (Heino and Godø 2002).

Most wild-living fish populations show large intraspecific variability in body shape, providing ample opportunity for natural or fisheries-induced selection to act on (Langerhans and DeWitt 2004). For example, gape-size-limited predators usually preferentially consume more slender individuals, creating selection pressures for humped body shapes (Brönmark and Miner 1992; Chivers et al. 2007). Harvesting through fishing may similarly generate size-independent selection differentials acting on morphological traits due to two major processes: (i) the physical interaction with the fishing gear and (ii) the potential covariation of body shape with other fitness-related traits. Indeed, due to the physics of the capture process in meshes, more streamlined fish tend to be selectively advantaged in gillnet fisheries because slender fish have a lower probability of retention in the nets than more humped individuals (Hamon et al. 2000). The physical interaction of fish with hooks is less studied compared to gill nets, but for fishing hooks to catch fish, hooks have to fit in the mouth of fish. With increasing hook sizes, progressively larger individuals are captured (Erzini et al. 1997; Alós et al. 2008; Cerdà et al. 2010), which should lead to selection pressures on small mouth gapes in heavily exploited fish species. Indeed, a larger vulnerability to fishing was documented for species that have larger mouth gapes, even when mouth size was corrected for variation in body size among individuals (Karpouzi and Stergiou 2003).

Besides this direct physical selection induced by hooks on aspects of morphology in fish, selection on morphotypes could also occur as a by-product when other traits are subjected to selection that covary with morphology. For example, intraspecific variability in body shape has been associated with different behavioral traits such as swimming behavior (Nilsson et al. 1995; Domenici and Blake 1997; Walker 1997; Andersson et al. 2006; Chivers et al. 2007; Pettersson 2007; Domenici et al. 2008), antipredator responses (Brönmark and Miner 1992; Nilsson et al. 1995; Domenici and Blake 1997; Walker 1997; Chivers et al. 2007; Domenici et al. 2008; Hulthén et al. 2014), habitat choice (Ehlinger 1990; Bourke et al. 1997), and adaptation to the local hydrodynamic conditions (Fulton et al. 2005; Langerhans 2008; Franssen 2011; Franssen et al. 2013; Binning et al. 2014). Because of the growing evidence suggesting that fishing using passive gears (where encounters of fish with gear depend on behavior) can generate strong selection differentials on behavioral traits (e.g., Biro and Post 2008; Uusi-Heikkilä et al. 2008; Nannini et al. 2011; Parsons et al. 2011; Wilson et al. 2011; Alós et al. 2012; Klefoth et al. 2012; Olsen et al. 2012; Sutter et al. 2012), such selection on behavior should indirectly create a selection differential on the associated morphology. For example, fish with larger swimming activity that also have a more elongated body (Andersson et al. 2006) should also have a larger probability to encounter a passively operated hook, which in turn should induce selection pressures on behavior and indirectly on morphology through a correlated response.

The objective of this study was to search for evidence for size-independent selection operating on fish body shape in a recreational marine fishery. We specifically tested whether recreational fishing is selective for certain body shapes in a field experiment in two harvested coastal fish species, *Diplodus annularis* and *Serranus scriba*. Our study is meant to be exploratory by first analyzing whether selection on morphological traits is conceivable in an intensive recreational fishery. Further work is reserved to more mechanistically understand any basis of morphological variation among the studied individuals in the wild.

## Materials and Methods

### Experimental setting

The basis of our approach was to compare the geometric body shape of individuals captured by hook-and-line recreational fishing (fished sample) and a random sample (population sample) of individuals from the population jointly sampled at the same locality and time. We focused on two commonly targeted coastal fish species, *Diplodus annularis* and *Serranus scriba*. These species are ecologically common in temperate coastal areas but are also among the most popular species targeted by the recreational fishery in the Mediterranean Sea (Alós and Arlinghaus 2013). Two experimental study sites in Palma Bay (Mallorca Island, Western Mediterranean) were chosen, each with a radius of 1000 m (see online supporting information Fig. S1). This area encompassed the average home range of the two species studied here (March et al. 2010, 2011). The location of the two study sites was selected according to the presence of suitable mesohabitat (*Posidonia oceanica* seagrass) and relatively low fishing and anthropogenic pressure so as to sample a rather natural assemblage.

To obtain the fraction of the population susceptible to hook-and-line gear, fishing sessions using recreational angling gear of 30-min duration were carried out in both study areas by volunteer anglers (accompanied by a researcher) following Alós et al. (2009). Natural bait (i.e., pieces of shrimp, *Penaeus vannamei,* of similar size and shape) was used, and sessions were performed from an anchored recreational boat in random places within the study area. To obtain the random sample of the population, we used an experimental active net designed for scientific assessment of fish assemblages inhabiting *P. oceanica* seagrass (see Moranta et al. 2006; Deudero et al. 2008). The active net was 3 m large and 1 m high and had a net body of 8 m (1.2-cm square mesh) and a 2-m-long cod end (0.6-cm square mesh). The experimental net was towed three times per site over the seagrass using a research vessel during 20 min for approximately 900 m. We assumed that the active net method would capture a greater fraction of the variation in body shapes present in the fish assemblage compared to the fished sample, and although all gears will be selective to some degree, this method allow the capture of a more random sample than possible with angling gear. Both samples were obtained during daytime.

**Figure 1:**
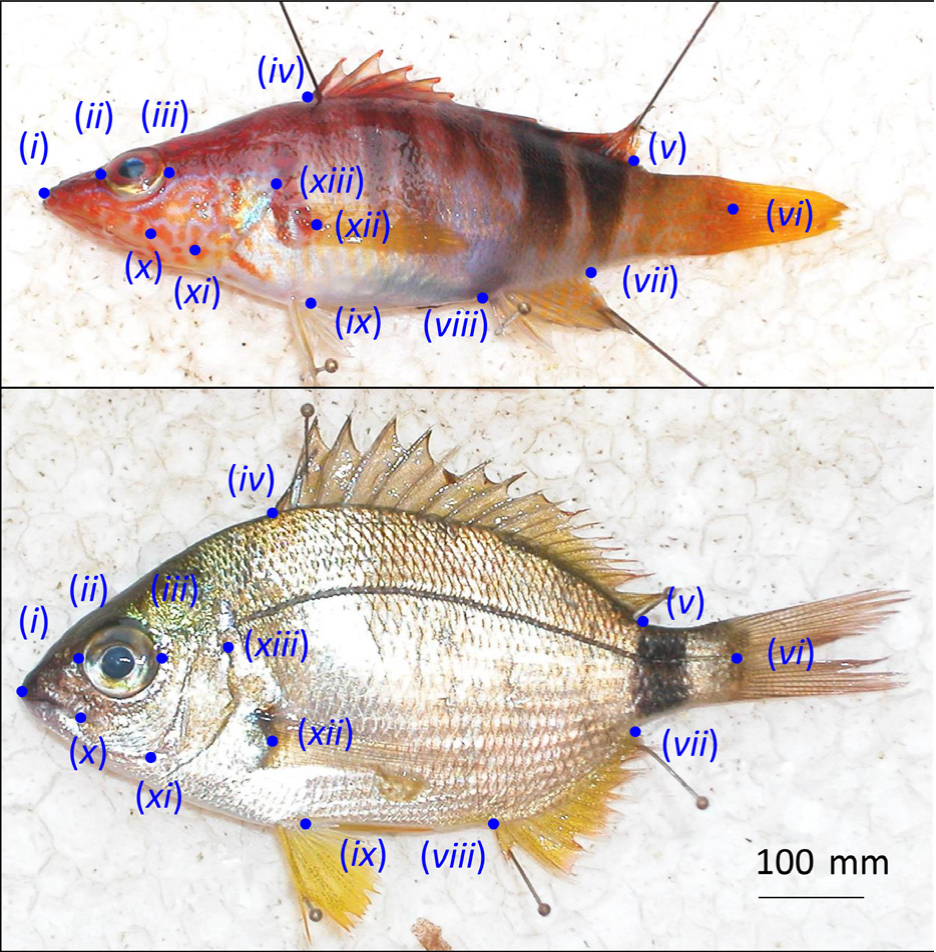
Body shape landmarks (*n* = 13) acquired in the two study species. The upper panel shows an individual of *Serranus scriba* and the down panel an individual of *Diplodus annularis*. In both cases, the coordinates (landmarks) acquired for this study are shown as blue points (labels from i to xiii).

A total of 473 individuals of *D. annularis* and 302 individuals of *S. scriba* were sampled with both gears. Fish were measured (total length, mm) and weighted (total weight, g), and a digital image of the left lateral size of each individual was taken using a digital camera (Olympus E300) (Fig. 1). We processed a subsample (*D. annularis*, *n* = 126 and *S. scriba*, *n* = 139) to confine the analysis to a narrow size range, that matched in both fishing gears. Although the mean size did not differ between the origin of the sample (see the electronic supporting information S2), limiting the body shape analysis to fish within the same narrow size interval controlled for possible allometric effects of size on body shape and ensured that our morphological results were size independent.

Fish with different condition due to variation in food resource intake are likely to have different body proportions influencing their body shape and hence our analysis (Einen et al. 1998). To account for systematic variation in fish condition of fish sampled with both methods (Huse et al. 2000), we calculated the relative condition index of the fish and it was used as covariate in the data analysis (see below). The index was calculated as the ratio between the observed weight and the predicted weight from an independently estimated length–weight relationship for both species of the area (Morey et al. 2003) following the protocol by Morgan (2004). The relative condition index was preferred because in contrast to Fulton's condition index, it is independent of body size (Morgan 2004). Controlling for size variation among gears and controlling body condition was done to remove any potential confounding effect on body shape and obtain a cleaner relationship of body shape and vulnerability to hook-and-line fishing gear.

### Quantification of body shape and data analysis

The body shape of each individual was analyzed using a landmark-based method (Rohlf and Marcus 1993). To that end, we selected 13 homologous landmarks (Fig. 1). The coordinates of these landmarks for each individual were acquired from a dorsal (left side) image of the fish using the tpsDig2 software (Rohlf 2004). The selected homologous landmarks were as follows: (i) tip of the upper jaw, (ii) anterior of the middle axis eye, (iii) posterior of the middle axis eye, (iv) anterior insertion of the dorsal fin, (v) posterior insertion of the dorsal fin, (vi) posterior extremity of the lateral line, (vii) posterior insertion of the anal fin, (viii) anterior insertion of the anal fin; (ix*)* insertion of the pelvic fin, (x) posterior corner of the upper jaw, (xi) corner of the pre-operculum, (xii) corner of the insertion of the pectoral fin, and (xiii) upper corner of the operculum. The raw coordinates were superimposed using general Procrustes superimposition (GPA) as implemented in the function *procGPA* from the *shapes* library (Dryden 2012) of the R package (R Development Core Team 2011). The superimposed coordinates were used as shape descriptors for further analyses.

**Figure 2:**
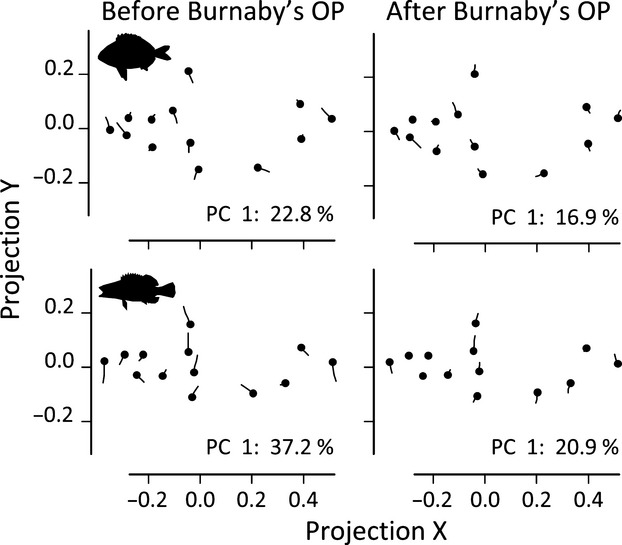
Approach to removing the arching effect following Valentin et al. (2008). Each panel represents the two extreme shapes of the main gradient of shape variation (first principal component axis (PC 1) of the shape descriptors; the amount of shape variability explained is indicated by a percentage). Each landmark of one of the two extreme shapes is indicated by points, and the other is represented by lines connecting the two shapes (note that is arbitrary which of the two shapes is represented by points). Before Burnaby's projection, the landmarks (i) and (vi) point toward one direction and the four central landmarks toward the opposite direction, thus suggesting that the fish is not correctly aligned but bent. After projection, (i) and (vi) point at opposite directions and, in the case of *S. scriba* (the two panels below), the four central landmarks suggest a deeper/compressed pattern.

Although specimens were carefully placed under the camera in a standardized way, both species suffered from some dorsoventral bending (Fig. 2). The shape differences associated with this bending would not represent true shape differences among gears (called *arching effect*, Valentin et al. 2008) and might obscure any shape patters. We removed the arching effect by projecting the shape descriptors onto a vector (Burnaby's orthogonal projection) that modeled the shape changes associated with bending following the method provided by Valentin et al. (2008). Figure 2 shows the results of applying such a protocol.

Arching-free shape descriptors were analyzed by conventional multivariate linear modeling. The response matrix (arching-free body shape) was constructed by the shape descriptors (columns) of each fish (rows). The explanatory variables were fish size and fish relative condition (continuous variables), study site (A and B, see Fig. 1S), and the origin of the sample (hook-and-line vs. random population). We also considered the interactions “fish size × study site” and “fish condition × study site.” The multivariate analysis was completed using the function *rda* as implemented in the *vegan* library (Oksanen 2005) of the *R* package. After removing (backward elimination) nonsignificant variables or interactions, the partial effects of the variables of interest were tested using a permutation approach. In addition, a linear discriminant analysis (LDA) was completed with the multivariate residuals after removing the effects of size, condition, and study site. The reliability of the differences between sampling methods inferred from LDA was checked via leave-one-out cross-validation. Finally, the shape corresponding to the averaged LDA scores for each one of the sampling methods was regressed on the arching-free shape descriptors for allowing an intuitive visualization and interpretation of the differences in body shape attributable to each of the gear samples (Monti et al. 2001; Linde et al. 2004).

A partial least squares (PLS) analysis was performed to explore whether different regions of the fish body varied independently (Klingenberg 2009). The PLS was performed considering two regions: (1) the head (landmarks i, ii, ii, x, xi, and xiii) and (2) the trunk (landmarks iv, v, vi, vii, viii, ix, and xii), within the configuration of the whole body, which takes into account not only shape changes between regions but also their topology and relative size relationships (Klingenberg 2009). The analysis was performed on size- and condition-corrected data and pooling within-group covariances by the origin of the sample and study site. The strength of the covariation between the two body regions was measured by the *RV*-coefficient (Klingenberg 2009). This coefficient varies from 0 to 1, where low values indicate that the regions vary independently and high values that they vary in a coordinate fashion. The reliability of the analysis was tested via a permutation test. A significant permutation test indicated that the *RV* value of the sample was higher than it would be expected by random chance alone; that is, the changes in the two regions would be correlated. PLS and permutation tests were completed using *MorphoJ* (Klingenberg 2011).

## Results

Multivariate linear regression of fish size, fish condition, study site, and sample origin (hook-and-line sample vs. random population) on body shape revealed that all of these explanatory variables had statistically significant effects in the case of *D. annularis* (Table 1, note that all interactions were nonsignificant and, therefore, they were not included in the final model). The origin of the fish sample explained a substantial fraction (29.6%) of the body shape variation among individuals (Table 1). The predictive capability of the discriminant analysis was high, with 76.7% and 84.8% of the individuals being correctly classified as being vulnerable to hook-and-line or constituting the random population sample, respectively. Similar results were obtained for *S. scriba,* for which body shape variation was also significantly correlated with the four variables evaluated (Table 1; note that interactions between the variables were again nonsignificant and were thus excluded from the final model). The origin of the sample explained the highest percentage of the body shape variation (34.7%) among all variables in *S. scriba* (Table 1). The predictive capability of the discriminant analysis in *S. scriba* was also high, with 73.4% and 76% of the individuals correctly classified to both fishing gears.

**Table 1:** Results of the redundancy multivariate analysis performed to test differences in the geometry of the body shape and the explanatory variables considered here for each of the species

Variable	Variance (×10^5^)	% variance	*F*	*Pr* (>*F*)
*Diplodus annularis*				
Fish size	7.77	48.88	12.08	<0.001***
Sample origin	4.701	29.58	7.31	<0.001***
Study site	1.927	12.12	3.00	<0.01**
Fish condition	1.497	9.42	2.33	<0.05*
*Serranus scriba*				
Sample origin	4.43	34.73	8.22	<0.001***
Fish size	3.238	25.39	6.01	<0.001***
Study site	3.109	24.38	5.77	<0.001***
Fish condition	1.977	15.50	3.67	<0.01**

Significant (*), highly significant (**) and very highly significant (***).

**Figure 3:**
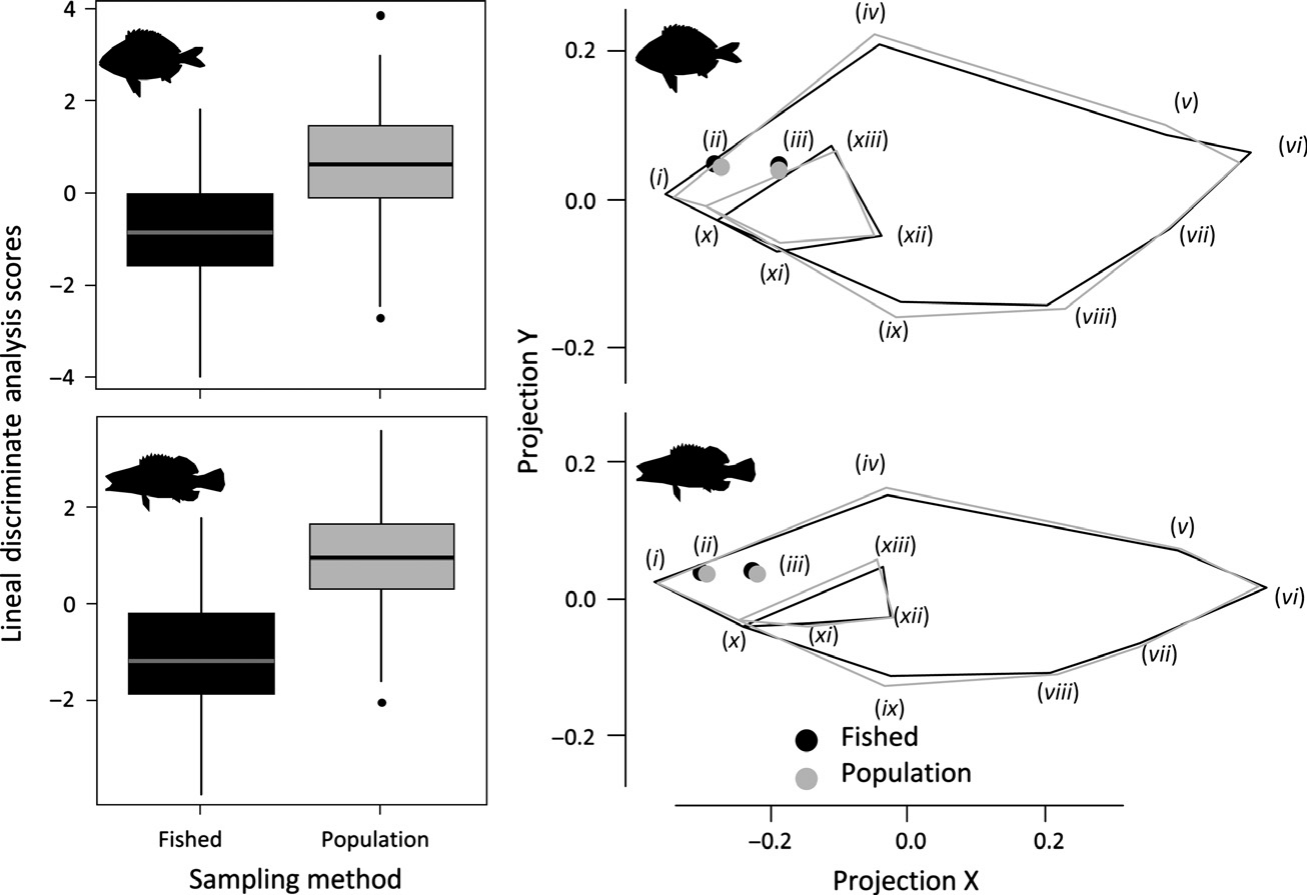
Box plots derived from the linear discriminant analysis (LDA) and the mean geometric body shape predicted for an average individual sampled either of the two methods: fished and population sample (note that these shape changes correspond to the sampling method only; the effects of “fish size,” “fish condition” and “study site” have been statistically removed). In both species, the main shape differences were localized at the mouth (landmarks i and x), the insertion of the dorsal and the pelvic fins (landmarks iv and ix) and the posterior extreme of the lateral line (landmark vi).

In both species, three key geometric body shape regions distinguished the average angled individual from the population (Fig. 3). First, the distance between the tip of the upper jaw (landmark i) and the anterior corner of the upper jaw (landmark *x*) (which was related with the mouth gape) was found to be larger for vulnerable individuals compared to the random population sampled by trawling (Fig. 3). Second, the distance between the anterior insertion of the dorsal fin (landmark iv) and the insertion of the pelvic fin (landmark ix) (which defines the body depth, that is, degree of streamlining) was smaller for the angled individuals, which indicates recreational fishing captured more streamlined and shallower fish compared to the trawled sample (Fig. 3). Third, analysis of the distance between the tip of the upper jaw (landmark i) and the posterior extremity of the lateral line (landmark vi) (which defines the general elongation of the body) revealed that the angled individuals were, on average, more elongated than the whole of the population (Fig. 3). These three patterns strongly suggested recreational fishing can induce a selection pressure on mouth shape and body shape. Note that these patterns were independent of size, fish condition, or study site (Fig. 3).

**Figure 4:**
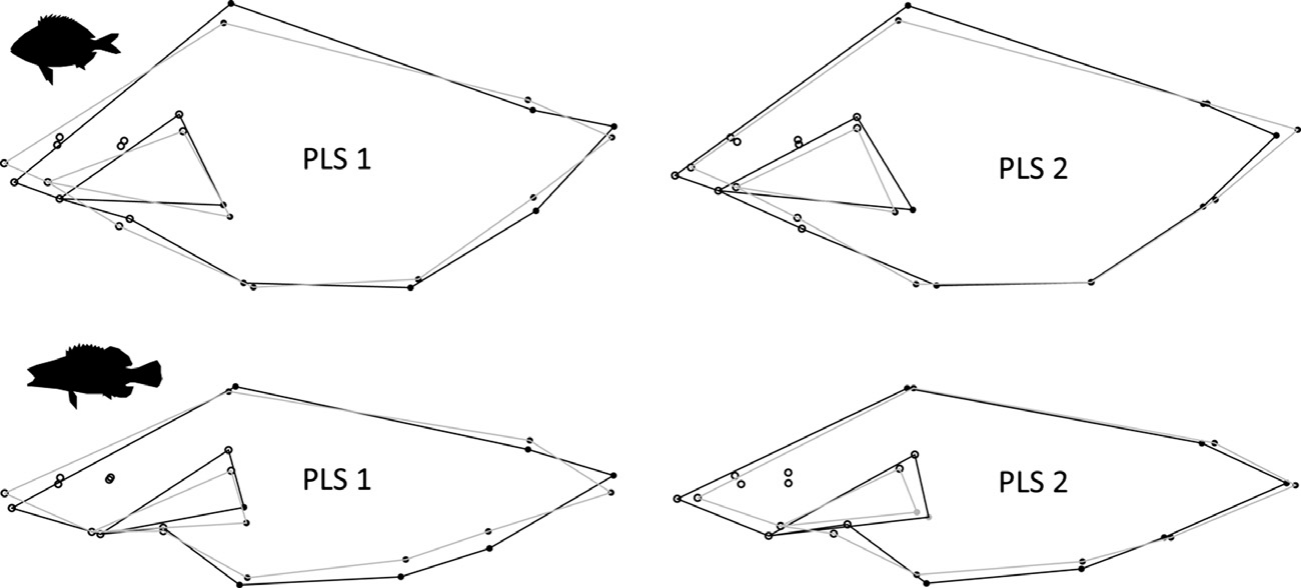
Patterns of covariation between the head and the trunk (light and solid black dots, respectively) in *D. annularis* (the two panels above) and *S. scriba* (the two panels below). The coordinates of the landmarks of the first and the second axis of the partial least squares (PLS 1 and 2) carried out for each species are represented. The maximum (black line) and the minimum (gray line) values observed for each axis of the PLS have been superimposed to improve visualization.

The PLS analysis revealed a weak pattern of covariation between the head and the trunk in both species. In *D. annularis*, the value of the *RV* coefficient was 0.344 (*P* < 0.001). The first PLS axis (variance explained 61.6%) depicted a pattern of covariation that involved the streamliness of the trunk and the position of the mouth: Deeper fishes also had a mouth in a more ventral position than shallower fishes (Fig. 4). The second PLS axis (23.9%) showed a relationship between both the streamliness and elongation of the trunk and the mouth gape: Deeper and shorter fishes had smaller mouths (landmarks i and ix) than streamlined and elongated fishes (Fig. 4). In *S. scriba*, the value of *RV* coefficient was 0.339 (*P* < 0.001). While the first PLS axis (68.0%) showed the same pattern of *D. annularis*, the second PLS (14.6%) axis showed that deeper and shorter individuals had larger mouths than shallower and elongated fishes (Fig. 4). These results suggested a relative high potential for independent selection of specific body shape regions by recreational fishing gear.

## Discussion

We found consistent empirical evidence across two exploited coastal fish species that recreational fishing is not a random mortality process in relation to body shape, while controlling for body size and condition variation. Specifically, individuals in the population of both species with larger mouths and more streamlined and elongated bodies were found to be more vulnerable to hook-and-line recreational fishing, in turn creating selection for smaller mouth and deeper bodies. Due to the only small degree of integration of these three different regions of the body, wild fish populations should show independent variation of these specific regions in response to selection by fishing. The direct physical interaction of a fish with the hooks can explain why similarly sized individuals with differential gapes can be expected to have a differential probability to get hooked. Body shape differences are also well known to be related to an individual's routine swimming behavior (Domenici and Blake 1997; Andersson et al. 2006; Langerhans and David 2010; Jones et al. 2013), which might also explain why we obtained selection differentials acting on morphology. Although body shape will also vary with resource intake (Parsons and Robinson 2007), our results of a clear relationship of morphology with vulnerability to angling were independent of individual variation in relative body condition or size. This is an important finding because it is theoretically possible that the more slender fish that were more vulnerable to fishing encompassed fish of a certain foraging mode in lower condition and hence in a state of elevated hunger, which is known to enhance catchability (Huse et al. 2000). Because our results held while controlling for condition, we favor the explanation that angling-induced selection on body shape could be related to direct selection pressures acting on correlated behavioral traits related to swimming behavior. Morphological integration is key trait for shaping a species evolutionary trajectory (Martínez-Abadías et al. 2012), and hence, fisheries-induced selection in morphology might have far-reaching consequences for the evolving species. Our work provides new insights into how the patterns of morphological integration can be used for understanding the selective properties of harvesting by identifying how different body regions exposed to selection can covary and can be altered by human.

The finding that fishing selects for fishes with larger mouth can, as mentioned before, most likely be attributed to the physical constraints emerging from the mouth gape of fish in relation to the size of the gear (hooks). For mere physical reasons, individuals with larger mouth areas will be more prone to ingest hooks or lures than individuals with small mouths (Lewin et al. 2006). The fact that vulnerability to fishing is determined by the physical interaction between the gear and the fish's morphology has been previously reported for gill nets and other mesh-based fishing gears (Reis and Pawson 1999; Hamon et al. 2000; Heino and Godø 2002; Stergiou and Karpouzi 2003). For example, Hamon et al. (2000) demonstrated how deeper-bodied sockeye salmon, *Oncorhynchus nerka*, had a higher probability of being entangled in the fishing nets; here the resulting fisheries-induced selection pressure acted in the opposite direction of sexual selection and predation-based natural selection pressures (Kendall and Quinn 2013). The novelty of our approach is that we provide evidence that hook-and-line fishing also selects on mouth morphology independent of the individual's body size or condition. Given the importance of the mouth morphology in facilitating the exploitation of foraging niches, which in some species is strongly involved in sympatric speciation (Wainwright 1988), fisheries-induced selection of mouth morphology may strongly alter predator–prey relationships and alter the evolutionary trajectory of exploited species. Therefore, larger mouth gapes may benefit individuals that are specialized to prey on large-bodied prey items, like *S. scriba* (Karpouzi and Stergiou 2003), and fishing selection on mouth size can affect negatively the foraging success and energy intake of surviving individuals.

We also found that a shallower and more elongated body had a higher vulnerability to be harvested by recreationally fished hooks. The physiological literature on fish swimming kinematics strongly supports the hypothesis that shallower and more elongated fish encompass more actively swimming individuals within a population (Brönmark and Miner 1992; Walker 1997; Andersson et al. 2006; Hanson et al. 2007; Langerhans and David 2010) as well as individuals that are more prone to continuous, long-distance swimming at larger swimming speeds (Domenici and Blake 1997; Walker 1997; Hanson et al. 2007; Langerhans and David 2010; Jones et al. 2013). Such behavior would increase the probability of encountering passive fishing gears (Rudstam et al. 1984; Kallayil et al. 2003; Biro and Post 2008; Løkkeborg et al. 2010; Alós et al. 2012) and consequently could explain the elevated vulnerability of shallow and elongated fish to angling gear as a correlated response of selection on behavior. By contrast, a deeper and more compressed body allows for better maneuverability than a streamlined and more elongated one (Domenici et al. 2008). Hence, a deeper-bodied morphotype is expected to display more tortuous searches for prey, often involving structured habitat such as that found in highly vegetated areas (Walker 1997; Pettersson 2007; Jones et al. 2013; Nash et al. 2013) with a smaller probability of encountering an angler (Alós et al. 2012). If morphology is correlated with behavior, our findings suggest that heavy exploitation by angling should drive exploited populations not only to become deeper but also to exhibit more tortuous foraging searches, less dispersal ability, and smaller activity spaces. Corresponding changes in life histories are possible (Alós et al. in press), but future research is needed to study the link of morphology–behavior and vulnerability to fishing to fully test the hypothesis that we introduce here based on morphological data alone.

Although there is large plasticity inherent on body shape (Langerhans and DeWitt 2004), variation in body depth among individuals has a significant genetic component (Toline and Baker 1997; Varian and Nichols 2010). Natural predation risk thus tends to not only plastically induce but also select for deeper bodies because this elevates handling time and reduces predation risk for surviving individuals (Brönmark and Miner 1992; Andersson et al. 2006; Domenici et al. 2008; Frommen et al. 2011). Similarly, deeper-bodied males are often favored by females in sexual selection and vica versa, presumably because this indicates a fitter individual (Hamon et al. 2000). Therefore, under high predation risk, natural and fisheries-induced selection may act in the same direction for this specific morphological region inducing more compact and deeper bodies in contrast to the mouth region where fishing and natural predators acts in the opposite direction. Hence, the interplay between natural and fishing selection, as well as the degree of integration of the fish body shape, does not lead to easy predictions as to how populations should develop morphologically in the presence of human exploitation, which is further complicated by the possibility of indirect selection on morphology through direct selection on behavior. However, if our hypotheses that body shape may serve as a proxy of underlying behaviors gains further support, we might have found a simple metric that might be used in large-scale comparative field studies to understand how differentially wild populations might respond morphologically and behaviorally to human and/or natural predation.

Three key messages can be derived from our work. First, fishing can select for a certain combination of morphological traits independent of body size. This selection process is likely due to two processes: direct selection caused by the physical features of the hooks relative to the size of the mouth and possibly as a by-product of direct selection on behavioral traits. The latter awaits further empirical analysis by studying behavior in the wild and linking behavior and morphology to vulnerability to fishing. However, collecting detailed spatial data by tracking wild animals in their free environment is technologically challenging (Krause et al. 2013), particularly in aquatic systems, and is thus unlikely to be available for large spatial scales. Thus, if our prediction on the relationship between body shape and behavior receives further support in other species and systems, body shape may emerge as a suitable surrogate for behavioral traits for studies on fisheries-induced phenotypic change. Second, fisheries-induced selection on morphological traits can produce strong selection responses over contemporary time scales due to the higher heritability of morphological traits compared to life history or behavior traits (Mousseau and Roff 1987; Roff 1992, 1997). Although the heritability of morphological traits will be species specific (e.g., Hard et al. 2008), a consistent selection on body shape could induce relatively fast genetic changes in exploited populations. Proper detection of fisheries-induced evolution is a key aspect of successful fish stock management, which requires continuous population monitoring (Kuparinen and Merilä 2007). Because of the simplicity of its assessment (relative to behavior or life history), body shapes could develop into simple metric in the study of fisheries-induced adaptive change, which may be easy in phenotypic time series over time. Finally, our work suggest a cautionary use of morphological information from sampled fish to infer population-level properties because of the potential sampling bias associated with samples collected with certain gears (fishery-dependent data, Ricker 1969). For example, passive sampling gear such as hooks or traps may produce bias in relation to inferring population-level morphological trait distribution from samples collected by angling exclusively. Likely, other gears suffer from the same limitation. We recommend more investigations to analyze how prevalent the selection of certain body shapes by different sampling methods is, particularly when comparing active and passive fishing methods.

## Data archiving statement

Data for this study are available in the Dryad repository: doi:10.5061/dryad.1k571

## Supporting Information

**Data S1.** Study site **Figure S1.** (A) Map showing the location of Mallorca Island (NW Mediterranean). **Data S2.** Sample size and test for fish size differences. **Table S1.** Sample size and parameters of a Generalized Lineal Model to test for differences in size of *Diplodus annularis* and *Serranus scriba* sampled and the origin of the sample (fished vs. population) and study sites.
