# Supplementary material for: Consistent size-independent harvest selection on fish body shape in two recreationally exploited marine species
Source: Ecol Evol. 2014 May 1;4(11):2154–64. doi: 10.1002/ece3.1075 (PMC4201430; doi:10.1002/ece3.1075)
Supplement: Supplementary file 1 [file ece30004-2154-sd1.doc]

Online supplementary material (study map, sample size and Generalized Lineal models of fish size)

for the manuscript

Consistent size-independent harvest selection on fish body shape in two recreationally exploited marine species

By

Josep Alós, Miquel Palmer, Marta Linde & Robert Arlinghaus

Contents:

*Online supplementary material S1: Study site*

*Online supplementary material S2: Sample size and test for fish size differences*

*Online supplementary material S1: Study site*


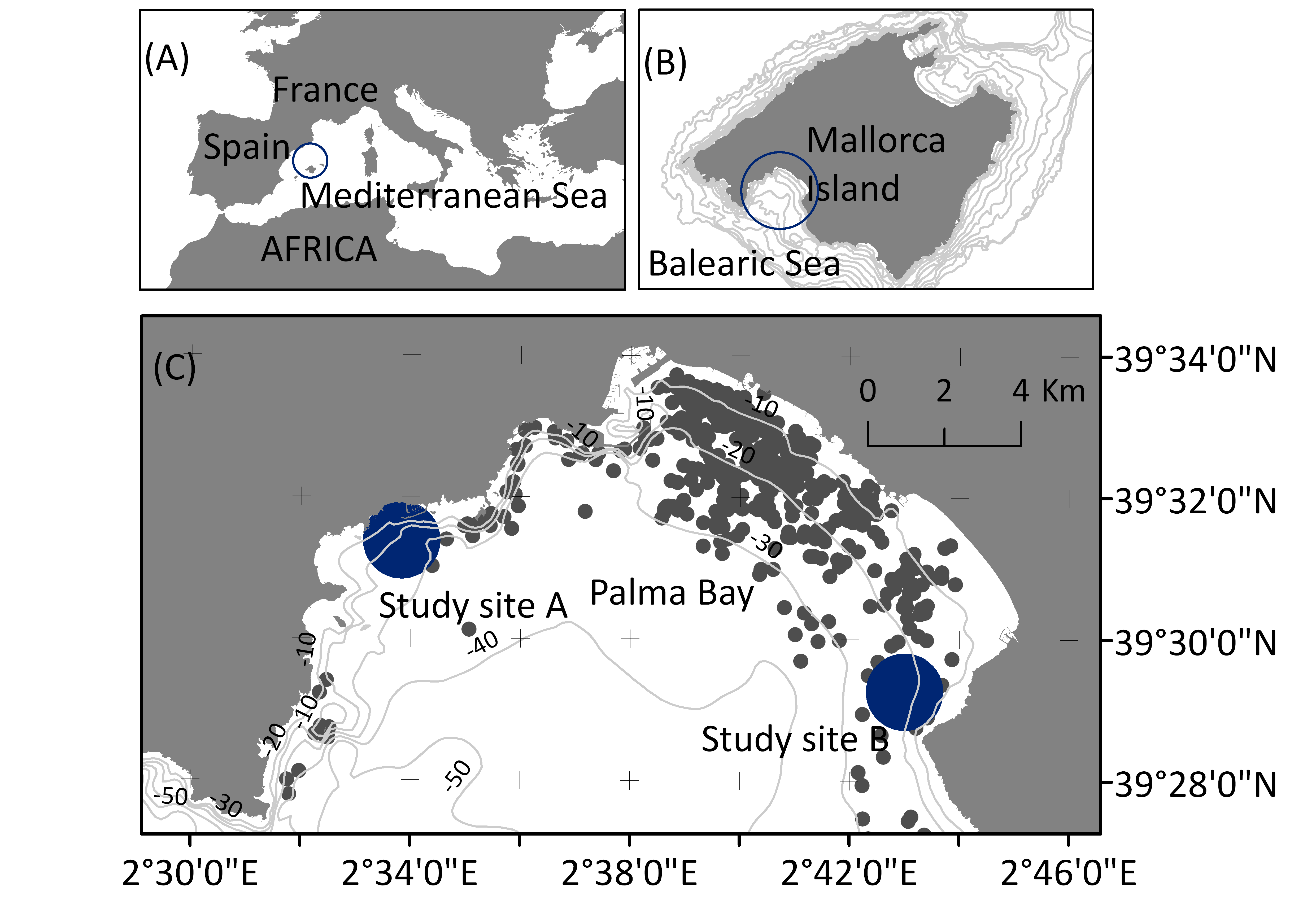


**Figure S1** (A) Map showing the location of Mallorca Island (NW Mediterranean). (B) Map showing the location of Palma bay where the experiment was carried out. (C) Map showing the two study sites (sites A and B). Study sites are marked in blue with a radius of 1,000 m where experimental sampling was performed. Each grey point corresponds to a recreational fishing boat fishing in Palma Bay censed during 70 visual random censuses performed in 2009 and 2010.

*Online supplementary material S2: Sample size and test for fish size differences*

|  | Sample origin | *Diplodus*  *annularis* (*n*=126) | *Serranus*  *scriba*  (*n*=139) |
| --- | --- | --- | --- |
|  |  | *n* | *n* |
| Study site A | Population sample | 30 | 33 |
|  | Fished sample | 28 | 30 |
| Study site B | Population sample | 36 | 42 |
|  | Fished sample | 32 | 34 |

|  | Estimate | Std. Error | *t*-value | Pr(>|t|) |
| --- | --- | --- | --- | --- |
| *Diplodus annularis* |  |  |  |  |
| (Intercept) | 86.13 | 3.42 | 25.19 | <0.001 |
| Sample (population) | 6.43 | 4.70 | 1.37 | 0.17 |
| Study site (A) | 8.55 | 5.01 | 1.71 | 0.09 |
| Sample (population) х Study site (A) | -9.38 | 6.92 | -1.36 | 0.18 |
| *Serranus scriba* |  |  |  |  |
| (Intercept) | 98.15 | 2.97 | 32.99 | <0.001 |
| Sample (population) | -0.08 | 4.00 | -0.02 | 0.99 |
| Study site (A) | 4.02 | 4.35 | 0.93 | 0.36 |
| Sample (population) х Study site (A) | -3.00 | 5.93 | -0.51 | 0.61 |

**Table S1** Sample size and parameters of a Generalized Lineal Model to test for differences in size of *Diplodus annularis* and *Serranus scriba* sampled and the origin of the sample (fished vs. population) and study sites. Note the absence of significant differences in all cases.
